# Cooperative Terrestrial-Underwater Wireless Optical Links by Using an Amplify-and-Forward Strategy †

**DOI:** 10.3390/s22072464

**Published:** 2022-03-23

**Authors:** Antonio Jurado-Navas, Carmen Álvarez-Roa, María Álvarez-Roa, Miguel Castillo-Vázquez

**Affiliations:** Wireless Optical Communications Lab., University Institute of Telecommunication Research (TELMA), University of Malaga, E-29071 Malaga, Spain; carmenalvarezroa@hotmail.es (C.Á.-R.); mariaalvarezroa@hotmail.es (M.Á.-R.); miguelc@ic.uma.es (M.C.-V.)

**Keywords:** combined terrestrial-underwater optical communication, amplify-and-forward, Málaga turbulence, Weibull

## Abstract

In this paper, we analyze a combined terrestrial-underwater optical communication link for providing high-speed optical connectivity between onshore and submerge systems. For this purpose, different transmission signaling schemes were employed to obtain performance results in terms of average bit error rate (ABER). In this sense, from the starting point of a known conditional bit-error-rate (CBER) in the absence of turbulence, the behavior of the entire system is obtained by applying an amplify-and-forward (AF) based dual-hop system: The first link is a terrestrial free-space optical (FSO) system assuming a Málaga distributed turbulence and, the second one, is an underwater FSO system with a Weibull channel model. To obtain performance results, a semi-analytical simulation procedure is applied, using a hyper-exponential fitting technique previously proposed by the authors and leading to BER closed-form expressions and high-accuracy numerical results.

## 1. Introduction

Optical communication systems are currently presented as a very competitive solution for the establishment of high capacity point-to-point wireless links, becoming an alternative [1,2,3] or, in the case of new generation networks [4,5,6,7], a complementary technology. This is the approach that has been included in the development of the 5G+ backbone systems infrastructure standard, which foresees the coexistence of millimeter radio technologies with terrestrial free-space optical (FSO) communications systems [8,9,10,11,12,13]. This growing interest in wireless optical communication technologies is mainly due to the enormous potential bandwidth inherent in these systems, which is much greater than that supported by radio systems, allowing very high binary regimes to be achieved without requiring any type of license [14]. On the other hand, given the enormous interest that has been aroused by everything related to the marine environment, justified by a multitude of reasons, both environmental and resource generation, the logical aim of extending telecommunication networks to the underwater environment has emerged. In this sense, the research community has focused the most recent efforts on the promotion of research and development into optical wireless communications systems in the underwater environment or underwater FSO [15,16,17,18,19].

Nevertheless, both channels present limitations in achievable performance imposed by the atmosphere and underwater transmission environments. First, in the case of terrestrial FSO systems, the main limiting agent is the atmospheric turbulence, which results in fluctuations in both the intensity and the phase due to the random inhomogeneities in the medium refractive index [20,21]. The effect of these inhomogeneities is known as scintillation, which involves random fades of the received optical signal intensity. To model this randomly fading characteristic of the atmospheric channel under variable turbulence conditions, extensive research has been performed by the scientific community. Accordingly, different mathematical models for the probability density function of the received irradiance have been proposed so far [22,23,24,25,26].

In the case of underwater FSO systems, the turbulent medium is the marine underwater environment but the effects over the optical signal are mainly the same, although originating from different physical phenomena. For example, the ocean dynamics that defines the turbulent process in the marine environment is mainly caused by ocean currents describing the motion of water within the oceans. Such currents induce temperature and pressure differences and, therefore, random fluctuations in its refractive index [27]. Factors such as turbidity [28] or salinity [29] of water are, in addition, completely related to refractive index variations, affecting the performance in these systems. In this respect, in order to characterize underwater optical turbulence-induced fading, underwater optical turbulence-induced fading can be described with different stochastic models such as the log-normal distribution [30], appropriated for weak fluctuation regime; the double Gamma model [29], accurately consistent with turbid seawater such as coastal and harbor water; or the Weibull function model, recently proposed in [18] to characterize the fading induced by either salinity or temperature in underwater optical channels.

The aim of this work is to extend the scope of optical wireless communications from the terrestrial to the underwater environment, thus inducing heterogeneous infrastructure that includes not only communication links in the atmospheric environment but also their extension to the underwater medium, by means of a cooperative terrestrial-underwater FSO (TU-FSO) system. Thus, the use of cooperative communications is naturally proposed as a consequence of enabling optical technologies in different media, both atmospheric and oceanic. In addition, cooperative transmission can significantly improve performance by increasing diversity order by using the most favorable relays from among the different nodes available in the communication network. This is a well-known technique in radio-frequency systems, where more attention has been directed to the concept of user cooperation as a new form of diversity for future wireless communication systems [31,32,33]. In the FSO communications context, cooperative links can be considered by assuming different techniques, such as parallel transmission schemes as well as serial transmission, evaluating bit error rate, outage probability and ergodic capacity when considering amplify-and-forward (AF) retransmission and decode-and-forward (DF) retransmission [10,34,35,36,37,38,39]. Among the different cooperative communication strategies, an AF link with variable gain is adopted in this work, allowing the evaluation of performance in terms of error rate when different block signaling schemes are assumed. Among the statistical distributions defined in the bibliography to model the scintillation effect induced by the medium turbulence, the Málaga or M [26] distribution has been adopted here for the terrestrial FSO link, whereas the Weibull distribution [18] has been selected to model the intensity fluctuations produced in the underwater FSO segment.

Then, in this work, the study of the cooperative TU-FSO system performance is analyzed in terms of average bit error rate (ABER) obtained under variable turbulence intensity conditions by applying a semi-analytical procedure. This method is based on the known closed-form expression of the conditional bit error rate (CBER) under the absence of turbulence effects, dependent on the signaling scheme adopted; namely, in this paper, uncoded on-off keying (OOK) has been used, as well as more complex OOK with memory coding techniques [40] and the variable-weight multiple pulse position modulation (vw-MPPM) scheme [41], both successfully applied in terrestrial FSO communications systems [42,43].

This paper is an extended version of our conference paper presented in [44] as invited speakers.

## 2. System Model

**Figure 1:**
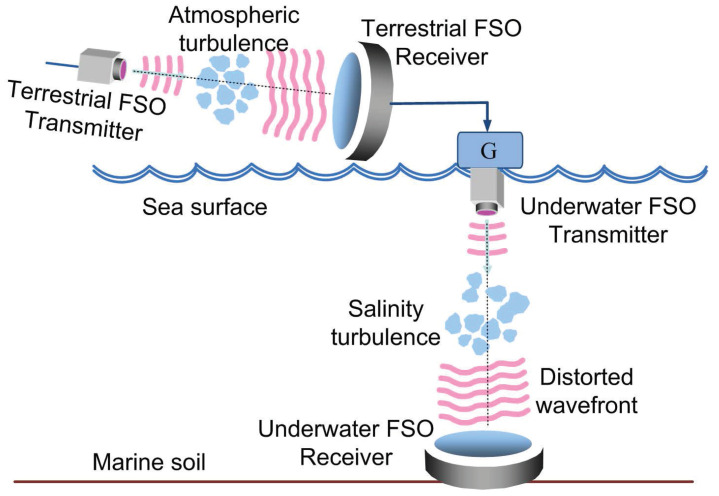
AF-based TU-FSO system model, based on the one in [44].

In this work, we analyze an amplify-and-forward (AF) based dual-hop TU-FSO system, as shown in Figure 1. In both hops, we consider point-to-point optical links using an intensity modulation with direct detection (IM/DD) scheme. The time-dependent photocurrent at the terrestrial FSO detector output is written as follows:

(1) $$i_{S_{1}}\left(t\right)=R_{1}h_{1}\left(t\right)P_{t}\left(t\right)+i_{N_{1}}\left(t\right),$$

where $R_{1}$ is the detector’s responsivity, with $h_{1}$ being the normalized ($E[h_{1}]=1$) scintillation affecting the irradiance, and following a Málaga (M) statistical distribution, as detailed in [26,45]. Furthermore, the average optical transmitted power is written as $P_{t}$, whereas it is assumed that the detector current noise signal, $i_{N}$, is mainly caused by a zero-mean additive white Gaussian noise (AWGN) with variance $\sigma_{n}^{2}$.

On the other hand, the terrestrial detector in the FSO link works as a relay node for cooperative communications. In this respect, it processes the received information data; then, such information is sent to the final destination. If this latter one is assumed to be the underwater FSO detector, then its time-dependent photocurrent can be then expressed as follows.

(2) $$i_{S_{2}}\left(t\right)=GR_{2}h_{2}\left(t\right)i_{S_{1}}\left(t\right)+i_{N_{2}}\left(t\right).$$

In (2), *G* is the channel state information (CSI)-assisted relay gain at the terrestrial FSO detector, as explained in [46], whilst $h_{2}$ denotes the normalized scintillation coefficient induced by the salinity and/or temperature gradient following a Weibull distribution. Moreover, parameters $R_{2},$ and $i_{N_{2}}$ were defined in the same sense as $R_{1}$ and $i_{N_{1}}$ were described for $i_{S_{1}}$, but now corresponding to the underwater channel. For the sake of simplicity, we have considered that the ocean ambient noise, $i_{N_{2}}$, is AWGN. Nevertheless, this assumption can be improved with the inclusion of an impulsive noise [47,48,49] described by the heavy tail rather than the exponential tail, since the AWGN model ignores the impulsive appearance of electromagnetic interference, oceanic noise or noises caused by humans using other machines, especially for scenarios with shallow water.

Without loss of generality, note that weather-induced attenuation has not been considered in this paper. Although that effect also degrades the performance of FSO systems in the manner shown in [50], however, due to its deterministic nature, such atmospheric attenuation acts merely as a scaling factor as indicated in [51]. Moreover, for analogous reasons, we have not considered the effect of absorption in water. As in the FSO link, absorption can be included as a pure loss term, as detailed in [52], acting as a scaling factor.

In addition, a comparison of underwater wireless optical communication system configurations and its associated performance can be found at [53]. For instance, for a laser diode wavelength of 450 nm employing OOK, a range of 20 m can be achieved for a data rate of 1.5 Gbps, as reported in [53]. More recently, a 100 m range through tap-water channel was demonstrated in [54], using OOK and a laser of 520 nm and 500 Mbps of data rate. Better performance can be obtained when employing a different modulation technique. Thus, as an illustrative example, in 2019, a commercial underwater optical communication product called BlueComm-200 was released, achieving a range of 150 m (>200 m for moderate to low turbidity dark water) with a data rate of 10 Mbps employing a 450 nm LED and a photomultiplier [55]. Finally, optical scattering [19,29,56] from suspended particles was incorporated in the model in a simple and straightforward manner. Such a scattering is largely independent of wavelength and depends on impurities and turbidity in the open water, and it results in a significant inter-symbol interference (ISI) (if the bit rate is not lowered to accommodate for temporal scattering) that limits seriously the performance of any underwater optical wireless communication system. Therefore, its effect is more pronounced in coastal areas than open oceans.

As detailed in [17], a single-input/single-output underwater optical wireless communication system is considered employing non-uniform OOK modulation with IM/DD due to its low cost and implementation simplicity. Let us suppose a green laser diode operating at 532 nm at the transmitter side and a circular receiver aperture at the receiver side. A direct consequence of this modulation scheme is that the input signal is non-negative as it is proportional to light intensity. Thus, the average optical power transmitted for any sequence of bits of information (each of them transmitted in a temporal pulse of a bit period of duration) is affected by the oceanic path loss and fading due to oceanic turbulence ($h_{2}$ in our system model). Then, a convolution operation of that sequence of pulses and a finite-length sequence of real coefficients denoting the equivalent discrete-time impulse response of the system is performed, resulting an additional summation term in (2) representing ISI, an effect associated with multipath interference. This latter interference is produced when an optical signal reaches the detector after encountering multiple scattering objects or multiple reflections from other underwater bodies. This eventually results in waveform time dispersion (time spreading) and decreases the data rate due to ISI. Nevertheless, the amount of multipath interference depends upon system specifications and the propagation environment. Hence, for shallow water environments, optical waves reflected from the surface or bottom generate multiple signals at the detector. However, for deep oceans, these surface and bottom reflections can be ignored [16]. Since it is coming from multipath underwater propagation, that ISI term (denoted by $i_{ISI}$), is also affected by $h_{2}$ in a multiplicative manner in the following manner.

(3) $$i_{S_{2}}\left(t\right)=GR_{2}h_{2}\left(t\right)i_{S_{1}}\left(t\right)+h_{2}\left(t\right)i_{ISI}\left(t\right)+i_{N_{2}}\left(t\right).$$

Namely, such an equivalent discrete-time impulse response of the system is constructed by the convolution of the transmitted filter, the fading free-channel impulse response with unity area and the employed matched filter. For the sake of simplicity, we model this ISI via its variance, $\sigma_{s}^{2}$. In Section 4, we describe the method for incorporating this ISI in a mathematically tractable manner to derive analytical expressions associated with the error probability of the complete TU-FSO system.

At this point, we want to remark that, in pure sea water or in clear oceans, initially, both absorption and turbulence will be limiting factors, and as the water approaches closer to the land, where organic matter and suspended particulates are present, scattering dominates. In this respect, a rate adaptive transmission scheme such as the ones analyzed in this manuscript can mitigate the adverse effect of the randomly changing underwater environment [57].

Additionally, we are aware that our chosen cooperative communication scheme (amplify and forward) requires the use of the same modulation format in air and undersea. Some free-space optical communication systems do not use OOK and either use DPSK, a bandwidth efficient coherent modulation, or photon efficient orthogonal modulations (such as PPM and FSK). However, most fielded undersea terminals (and the ones currently purchasable) implement OOK at 1 GHz or lower due to limitations in available COTS (commercially available off-the-shelf) components in blue–green. It is, therefore, likely that any relay that wants to communicate between aerial and undersea systems would need a modulation format change. In the TU-FSO system presented in this work, we have avoided that requirement for most of the cases analyzed since the FSO system operates directly with either OOK or rate-adaptive transmission schemes based on the aforementioned OOK.

To complete this section and for the sake of simplicity, we assume that the terrestrial FSO receiver is steady on the marine surface (for example, it is fixed on an oil platform), avoiding sea movements that would seriously affect the performance of the aforementioned terrestrial FSO receiver. In a general scenario, the stability of the terrestrial FSO receiver would become an important factor for the performance of the entire system, and the resulting adverse effect would be described by misalignment and angular pointing errors. All in all, underwater wireless optical communication systems are less sensitive to angular pointing errors due to jitter since scattering is able to alleviate such a fading effect at the expense of a higher attenuation due to geometric spread, as detailed in [58].

## 3. Channel Model

**Figure 2:**
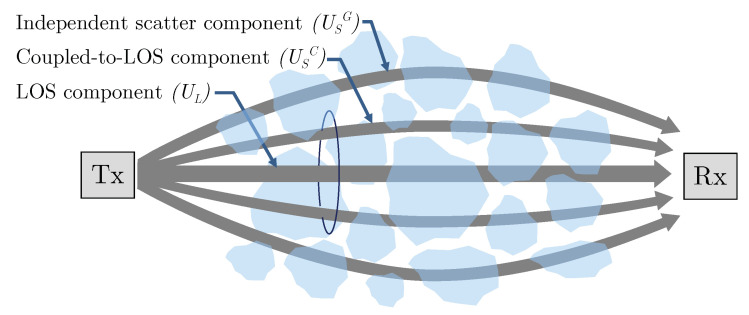
Laser beam propagation scheme under an M-distributed FSO link [45].

In this paper, we consider a M probability density function (PDF) to model atmospheric scintillation, $h_{1}$ [26]. Thus, $h_{1}$ is described by a modulation process as a product of two stationary random terms: one arising from large-scale turbulent eddy effects, mainly due to refractive effects; and the other one representing the small scale atmospheric fading characteristic, which is primarily due to diffraction effects. Accordingly, Figure 2 shows its associated small-scale laser propagation scheme. There, the observed field at the receiver is supposed to consist of three terms: the line-of-sight contribution $U_{L}$; a new scattering component, $U_{S}^{C}$, that is quasi-forward scattered on the propagation axis and assumed to be coupled to $U_{L}$; and, in addition to the classical scattering optical field, $U_{S}^{G}$, due to energy that is scattered to the receiver by off-axis eddies. A detailed description of this model can be found in [26].

Precisely, following [26], the model defines some other parameters to completely characterize the received irradiance fluctuation. Thus, parameter $\Omega =E[U_{L}^{2}]$ represents the LOS average optical power, whereas $\xi =E[|U_{S}^{C}{|}^{2}+|U_{S}^{G}{|}^{2}]=\xi_{c}+\xi_{g}$ denotes the average power of the total scattering components: either the coupled-to-LOS and the classic one, respectively. On another note, the relationship between those two scattering components is described by parameter ρ, representing the amount of scattering power coupled to the LOS component, with 0≤ρ≤1. Hence, their average powers are, respectively, $\xi_{c}=\rho \xi$ and $\xi_{g}=\left(1-\rho \right)\xi$, and the total average optical power is given by $E[h_{1}]=\Omega +\xi$.

In addition, as detailed in [45], the Málaga PDF can be reformulated by involving a mixture of continuous Generalized-K and a discrete Binomial distributions. This fact results in a novel and interesting physical interpretation of the M statistical model, where the optical channel are considered as a superposition of different independent subchannels described by a generalized-K PDF, $K_{G}$, with each subchannel with a different probability and $m_{k}$ is associated with a Binomial PDF. In this respect, the received irradiance PDF can be written as follows:

(4) $$f_{h_{1}}\left(h_{1}\right)=\sum_{r=1}^{\beta }m_{r}K_{G}\left(h_{1};\alpha ,r,I_{r}\right),$$

where $m_{r}$ is the probability that the optical signal travels through the r-th optical path, with α being a positive parameter related to the effective number of large-scale cells of the scattering process and β∈N being the shape parameter of the Nakagami-m distribution related to the slow fluctuation of the line-of-sight component, as discussed in [26]. Moreover, $I_{r}$ represents the mean optical irradiance of the *r*-th Generalized-K term. On the other hand, the large-scale effects are assumed to be common for every small-scale subchannel. Namely these small-scale subchannels are governed by small-scale diffraction effects, with the lower subchannel orders referring to more adverse turbulence regimes.

On a different matter and following the works introduced in [18,59], we employ a Weibull function model to characterize the fading associated with salinity-induced turbulent underwater channels. For this case, the Weibull model offers an excellent accuracy when it is utilized to predict experimental data, becoming particularly interesting in transition areas between seas and oceans where different masses of water meet. Furthermore, it provides a good fit when the receiver aperture is much larger than the coherence radius of the medium, which is something feasible considering the short-range links involved in underwater FSO systems. Mathematically, its PDF is expressed as follows:

(5) $$f_{h_{2}}\left(h_{2}\right)=\frac{K}{\lambda }{\left(\frac{h_{2}}{\lambda }\right)}^{K-1}\;\exp\left[-{\left(\frac{h_{2}}{\lambda }\right)}^{K}\right],$$

with K>0 being the shape parameter related to the scintillation index of the irradiance fluctuations and λ>0 is the scale parameter related to the mean value of the irradiance [60]. Assuming $E[h_{2}]=1$, then, according to [61], $\lambda =\frac{1}{\Gamma (1+1/K)}$, whereas the scintillation index is provided by $\sigma_{h_{2}}^{2}=\frac{\Gamma (1+2/K)}{\Gamma {\left(1+1/K\right)}^{2}}-1\approx K^{-11/6}$.

Once we have presented the statistical PDFs associated with either the atmospheric and underwater channels, now we can use that information to write the expression of the cumulative distributive function (CDF) for the CSI-assisted AF-based TU-FSO system. Thus, we have the following.

(6) $$F_{\gamma }\left(\gamma \right)\approx \mathrm{Pr}\left[\gamma_{1}<\gamma ⋃\gamma_{2}<\gamma \right]=F_{\gamma_{1}}\left(\gamma \right)+F_{\gamma_{2}}\left(\gamma \right)-F_{\gamma_{1}}\left(\gamma \right)F_{\gamma_{2}}\left(\gamma \right).$$

In this last expression, $\gamma_{1}$ and $\gamma_{2}$ are the signal-to-noise ratios (SNR) corresponding to the terrestrial and the underwater FSO hops, respectively. Their associated CDFs are directly obtained by integrating their PDFs, which are shown in Equations (4) and (5), respectively, as follows:

(7) $$F_{\gamma_{1}}\left(\gamma_{1}\right)=\sum_{r=1}^{\beta }\frac{m_{r}}{\Gamma (\alpha )\Gamma (r)}{\left(\frac{\gamma B}{\bar{\gamma_{1}}}\right)}^{\frac{b-a+1}{2}}\;G_{1,3}^{2,1}\left(\frac{B\gamma }{\bar{\gamma_{1}}}|\begin{array}{c} \frac{a-b+1}{2}\\ a,0,\frac{a-b-1}{2} \end{array}\;\right)$$

(8) $$F_{\gamma_{2}}\left(\gamma_{2}\right)=1-\exp\left[-{\left(\frac{\gamma }{\bar{\gamma_{2}}\lambda }\right)}^{K}\right],$$

where we have employed [62] (Eq. (6.592.2)) to obtain the CDF of the M model. In Equations (7) and (8), $\bar{\gamma_{1}}$ and $\bar{\gamma_{2}}$ correspond to average SNRs of atmospheric and underwater fading channels, respectively. In addition, a=α−r, b=α+r−1 and $B=\frac{\alpha \beta }{\Omega^{{}^{\prime }}+\xi_{g}\beta }$, with $\Omega^{{}^{\prime }}=\Omega +\xi_{c}+2\sqrt{\Omega \xi_{c}}$ representing the average power from the coherent contributions.

Hence, by inserting (7) and (8) into (6), we obtain the resulting overall CDF.

(9) $$F_{\gamma }\left(\gamma \right)=1-\;\exp\left[-{\left(\frac{\gamma }{\bar{\gamma_{2}}\lambda }\right)}^{K}\right]\times \left\{1-\sum_{r=1}^{\beta }\frac{m_{r}}{\Gamma (\alpha )\Gamma (r)}\;{\left(\frac{\gamma B}{\bar{\gamma_{1}}}\right)}^{\frac{b-a+1}{2}}\;G_{1,3}^{2,1}\left(\frac{B\gamma }{\bar{\gamma_{1}}}|\begin{array}{c} \frac{a-b+1}{2}\\ a,0,\frac{a-b-1}{2} \end{array}\;\right)\right\}$$

## 4. Performance Analysis

In this section, we derive the analytical expression for the ABER associated with the cooperative TU-FSO system described here. Considering the CDF shown in (9), a new highly accurate closed-form expression can be obtained for the ABER of an AF-based dual-hop TU-FSO system under all regimes of turbulence strength when employing any generalized coding technique. We will distinguish two scenarios: first, one where we can deduce an analytical closed-form expression for its associated conditional BER (CBER) of a particular signaling technique; in this case, we have provided the case of a classic uncoded OOK format that additionally can be used as a reference. Moreover, as a second scenario, we consider any generic coding scheme for which its closed-form analytical expression for CBER is unknown and, thus, closed-form expressions for the ABER cannot be easily derived. For this latter case, a curve-fitting method based on a hyper-exponential fitting technique introduced in [43] has been applied to derive a highly accurate mathematically tractable expression for CBER, which can then be applied to the procedure here described to obtain a closed-form expression for ABER.

### 4.1. Uncoded OOK Scheme

Since the most basic form of pulsed modulation in digital communications is OOK, we propose its analysis as a typical example of a coding technique for which its CBER can be analytically derived. The firstly calculated CBER for a given electrical signal-to-noise ratio in absence of optical turbulence, $\gamma_{0}$, represents the previous step for obtaining the final ABER featuring the system. From [22], the CBER of IM/DD with AWGN channel using OOK is expressed as follows:

(10) $$P_{b}\left(e|h\right)=\frac{1}{2}\mathrm{erfc}\left(\frac{i_{S_{0}}h}{2\sqrt{2(\sigma_{n}^{2}+\sigma_{s}^{2})}}\right),$$

with ${i_{S}}_{0}=attRP_{t}$ denoting the signal current in the absence of turbulence-induced fading, with att, *R* and $P_{t}$ representing the attenuation coefficient associated with the medium (not considered in this paper), the responsivity and the average of transmitted optical power, respectively. In (10), $\sigma_{s}^{2}$ represents the variance associated with the ISI affecting the system in the optical underwater channel, as commented in Section 2. There, it was described how the ISI term is affected by $h_{2}$, $\sigma_{s}^{2}\left(h_{2}\right)$, since it results from multipath interference and its inherent waveform time dispersion. Following [63,64], we have assumed in (10) that the ISI interference is Gaussian distributed.

Next, we define the signal-to-interference-plus-noise ratio (SINR) as follows:

(11) $$\gamma_{0}=\frac{{i_{S}}_{0}}{\sqrt{\sigma_{n}^{2}+\sigma_{s}^{2}\left(h_{2}\right)}},$$

where $\sigma_{s}^{2}\left(h2\right)$ represents the dependency of $\sigma_{s}^{2}$ on $h_{2}$. Now, to solve the integral involving ABER, calculated by averaging $P_{b}\left(e|h\right)$ over the PDF of the irradiance, $f_{h}\left(h\right)$, the SINR in (11) can be approximated by averaging the noises and the inter-symbol interference over oceanic turbulence in the way proposed in [64].

(12) $$\gamma_{0}\approx \frac{{i_{S}}_{0}}{\sqrt{<\sigma_{n}^{2}>+<\sigma_{s}^{2}\left(h_{2}\right)>}}.$$

In (12), <·> denotes the average over turbulence. Thus, and for the sake of consistency with notation in Equation (9), we can identify $\gamma =\gamma_{0}h$, with *h* representing the random normalized irradiance fluctuation. Therefore, the ABER, $P_{b}\left(e\right)$, is calculated by averaging $P_{b}\left(e|h\right)$ over the PDF of the irradiance, $f_{h}\left(h\right)$. Hence, we have the following.

(13) $$P_{b}=\int_{0}^{\infty }\frac{1}{2}\mathrm{erfc}\left(\frac{\gamma_{0}h}{2\sqrt{2}}\right)f_{h}\left(h\right)dh.$$

In (13), the PDF of the optical irradiance is defined according to the combined Málaga–Weibull model, as detailed in the previous section. Following [65], the ABER, $P_{b}\left(e\right)$, is obtained by averaging CBER over the CDF of *h*, $F_{h}\left(h\right)$, by using the integration by parts with the following formula.

(14) $$P_{b}=(P_{b}\left(e|h\right)F_{h}\left(h\right)){|}_{0}^{\infty }-\int_{0}^{\infty }\frac{d}{dh}\left[P_{b}\left(e|h\right)\right]F_{h}\left(h\right)dh.$$

Since $P_{b}\left(e|\infty \right)=0$ and $F_{h}\left(0\right)=0$ (note that negative values for the optical irradiance are not allowed), then the latter expression can be reduced to the following:

(15) $$\begin{array}{cc} P_{b}= & -\int_{0}^{\infty }\frac{d}{dh}\left[P_{b}\left(e|h\right)\right]F_{h}\left(h\right)dh=-\int_{0}^{\infty }\frac{d}{dh}\left[\frac{1}{2}\mathrm{erfc}\left(\frac{\gamma_{0}\cdot h}{2\sqrt{2}}\right)\right]F_{h}\left(h\right)dh, \end{array}$$

where $F_{h}\left(h\right)$ is directly obtained in (9). Now, we apply [66] (Eq. (06.27.13.0005.01)) to derive an expression for the derivative of $P_{b}\left(e|h\right)$ with respect to *h*.

(16) $$\frac{d}{dh}\left[P_{b}\left(e|h\right)\right]=-\frac{\gamma_{0}}{2\sqrt{2\pi }}\exp\left[-{\left(\frac{\gamma_{0}h}{2\sqrt{2}}\right)}^{2}\right].$$

Next, we introduce (16) in (15) in order to solve the resulting integral. For this aim, a generalized Gauss–Laguerre quadrature [67] is proposed, and it is defined by the following:

(17) $$\int_{0}^{\infty }x^{\upsilon }e^{-x}f\left(x\right)dx=\sum_{i=1}^{n}H_{i}f\left(x_{i}\right)+E_{n},$$

where υ is a constant, $x_{i}$ represents the i-th zero of the Laguerre polynomial, $L_{n}^{\upsilon }\left(x\right)$, $H_{i}$ is the corresponding weight coefficients associated with the Gauss–Laguerre quadrature and $E_{n}$ denotes the truncation error. If the normalization of the Laguerre polynomials is chosen such that the following is the case:

(18) Lnυ=∑m=0nn+υn−m(−x)mm!,

then, according to [67], the weight coefficients are provided by the following.

(19) $$H_{i}=\frac{\Gamma \left(n+\upsilon +1\right)x_{i}}{n!{\left(n+1\right)}^{2}{\left[L_{n+1}^{\upsilon }\left(x_{i}\right)\right]}^{2}},\;\left(i=1,2,\ldots ,n\right).$$

If the following change of variables is performed at the following point:

(20) $$x={\left(\frac{\gamma_{0}}{2\sqrt{2}}\right)}^{2}h^{2};\;dx=2{\left(\frac{\gamma_{0}}{2\sqrt{2}}\right)}^{2}hdh;$$

then we can apply (17) to solve (15). In this manner, we identify υ=−1/2; thus, we obtain the following:

(21) $$P_{b}=\frac{1}{2\sqrt{\pi }}\sum_{i=1}^{n}\;H_{i}F_{\gamma }\left(\gamma_{i}\right){|}_{\gamma_{i}={\left(2\sqrt{2}x_{i}^{1/2}\right)}^{\prime }}$$

where, again, $\gamma_{i}=\gamma_{0}\cdot h$ and $\gamma_{0}\approx {i_{S}}_{0}/\sqrt{<\sigma_{n}^{2}>+<\sigma_{s}^{2}\left(h_{2}\right)>}$, with $F_{\gamma }\left(\gamma_{i}\right)$ being the CDF proovided in Equation (9).

### 4.2. Coded Transmission Schemes

The second scenario considered in this section consists of any generic coding scheme for which its closed-form analytical expression for CBER is unknown, although accurately estimated by a curve-fitting method. In this respect, signaling techniques such as OOK-GScc [40,42] and vw-MPPM [41] can be included as representative examples belonging to this group: OOK-GScc is a Markov-chain-based coding scheme that can be studied as a run-length limited (RLL) sequences generator, with the great advantage that the associated decoder can be built by means of a shift register and simple combinational logic, which drastically reduces the decoding complexity if compared to the classical scheme based on the Viterbi algorithm. OOK-GScc provides a native coding rate of 1/4 and, to provide additional coding rates, the procedure consists in introducing a selective number of silence periods behind each coded bit generated by the OOK-GScc scheme. In this paper, effective coding rates of 1/4 and 1/8 are employed in the analysis. On the other hand, the vw-MPPM coding scheme is a nonlinear block coding technique based on translation table between input data codewords and output codewords, with the main goal of increasing the peak-to-average optical power ratio (PAOPR), maintaining the average optical power transmitted to the medium constant. For these coding methods, to the knowledge of the authors, there is no closed-form expression for BER under a simple additive white Gaussian noise (AWGN) channel; thus, they are appropriate for applying the curve-fitting method based on a hyperexponential fitting technique [43], obtaining the following analytical approximate equation:

(22) $$\mathrm{CBER}\left(h,\gamma_{0}\right)=P_{b}\left(e|h\right)\approx a_{c}\exp\left[-b_{c}{\left(\gamma_{0}^{2}h^{2}\right)}^{c_{c}}\right]$$

with $\gamma_{0}$ being the electrical SINR in absence of turbulence defined in (12) and where the hyperexponential fitting parameters are $a_{c},b_{c},c_{c}\in {ℜ}^{+}.$

These three parameters depend on the used coding scheme and are calculated by employing a least-squares fitting method over the results of BER accomplished by Monte-Carlo simulations without the effect of the turbulence process. Table 1 shows the resulting fitting parameters for OOK-GScc, with extended coding rates of R=1/4 and 1/8, and the vw-MPPM coding schemes calculated in [43].

By applying the same steps as in the previous section to simplify (14), i.e., applying $P_{b}\left(e|\infty \right)=0$ and $F_{h}\left(0\right)=0$, and considering that negative values for the optical irradiance are not allowed, the ABER, $P_{b}\left(e\right)$, is obtained by averaging CBER over the PDF of *h* by using the equivalent integration by parts formula:

(23) $$P_{b}=-\int_{0}^{\infty }\frac{d}{dh}\left[P_{b}\left(e|h\right)\right]F_{h}\left(h\right)dh$$

where the derivative of (22) with respect to *h* is obtained as follows.

(24) $$\frac{d}{dh}\left[P_{b}\left(e|h\right)\right]=-2a_{c}b_{c}c_{c}\cdot \gamma_{0}^{2c_{c}}h^{2c_{c}-1}\exp\left[-b_{c}{\left(\gamma_{0}h\right)}^{2c_{c}}\right].$$

Next, a generalized Gauss–Laguerre quadrature is again applied, as shown in (17), and with the help of (18) and (19). Then, inserting (24) into (23), comparing this latter one with (17) and identifying terms, we can directly obtain the following:

(25) $$P_{b}=a_{c}\sum_{i=1}^{n}\;H_{i}F_{\gamma }\left(\gamma_{i}\right){|}_{\gamma_{i}={\left(\frac{x_{i}}{b_{c}}\right)}^{{1/(2c_{c})}^{\prime }}}$$

where $H_{i}$ represents the weight coefficients and $x_{i}$ is the i-th zero of the Laguerre polynomial. Again, $\gamma_{i}=\gamma_{0}\cdot h$, with $\gamma_{0}$ written in (12), whereas $F_{\gamma }\left(\gamma_{i}\right)$ is the CDF calculated in Equation (9).

## 5. Results

**Figure 3:**
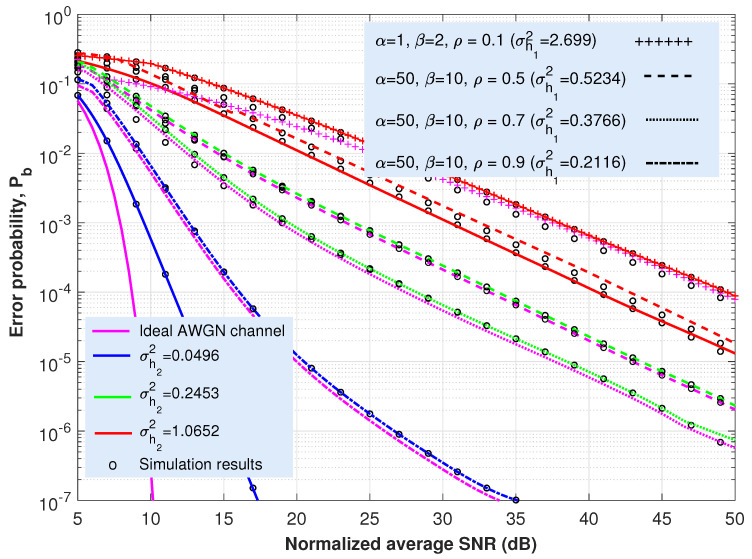
Analytical average bit error rate (ABER) and Monte Carlo simulation results (circles) vs. SINR $\gamma_{0}$ for a conventional OOK format under weak ($\sigma_{h_{2}}^{2}=0.0496$), weak-to-moderate ($\sigma_{h_{2}}^{2}=0.2453$) and strong ($\sigma_{h_{2}}^{2}=1.0652$) salinity induced turbulence and different Málaga turbulence intensities in the terrestrial FSO link. Scenarios and values of irradiance variances in the underwater medium are taken from acquired data presented in [68]. In solid lines, the performance of the system when only oceanic turbulence is considered is represented. As a reference, the ideal AWGN channel is depicted with a solid line.

**Figure 4:**
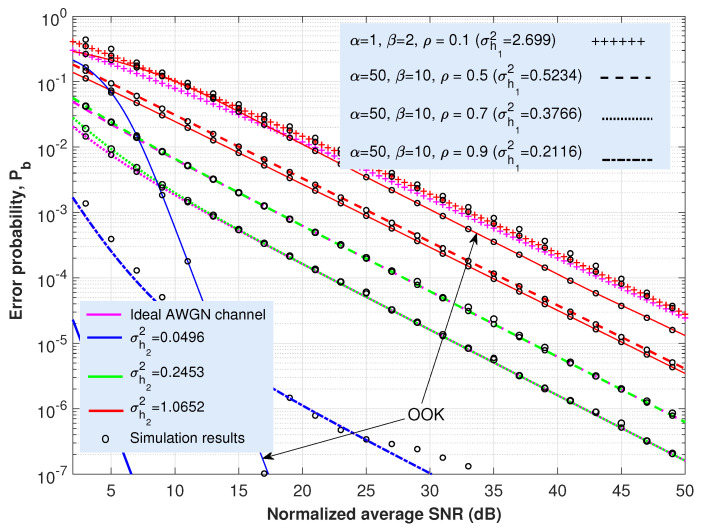
Analytical ABER and Monte Carlo simulation results (circles) vs. SINR $\gamma_{0}$ for an OOK-GScc format with code rate of 1/4 under weak ($\sigma_{h_{2}}^{2}=0.0496$), weak-to-moderate ($\sigma_{h_{2}}^{2}=0.2453$) and strong ($\sigma_{h_{2}}^{2}=1.0652$) salinity-induced turbulence and different Málaga turbulence intensities in the terrestrial FSO link. Scenarios and values of irradiance variances in the underwater medium taken from acquired data presented in [68]. In solid lines, the performance of the system when only oceanic turbulence is considered is represented.

**Figure 5:**
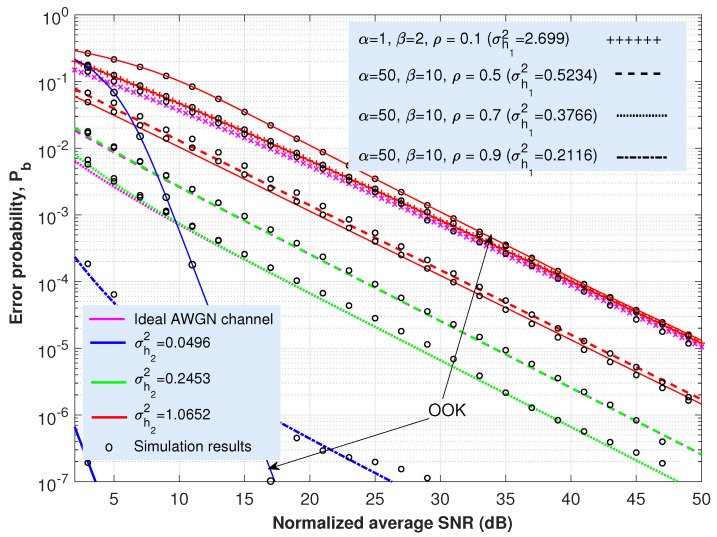
Analytical ABER and Monte Carlo simulation results (circles) vs. SINR $\gamma_{0}$ for an OOK-GScc format with a code rate of 1/8 under weak ($\sigma_{h_{2}}^{2}=0.0496$), weak-to-moderate ($\sigma_{h_{2}}^{2}=0.2453$) and strong ($\sigma_{h_{2}}^{2}=1.0652$) salinity induced turbulence and different Málaga turbulence intensities in the terrestrial FSO link. Scenarios and values of irradiance variances in the underwater medium taken from acquired data presented in [68]. In solid lines, the performance of the system when only oceanic turbulence is considered is represented.

**Figure 6:**
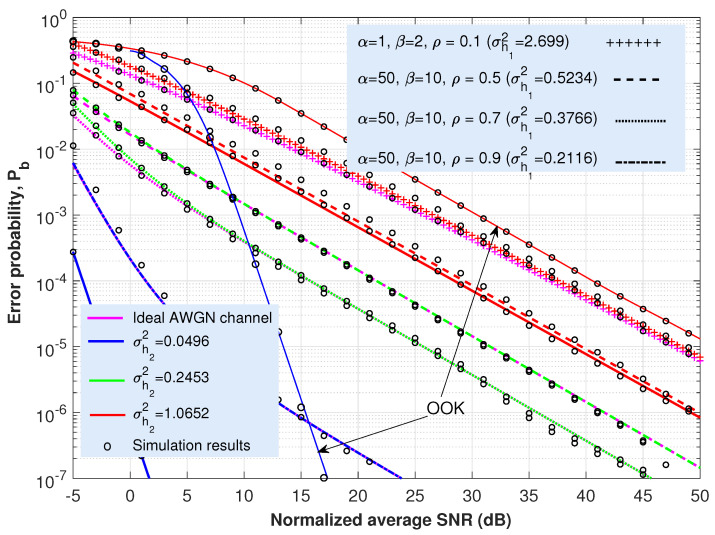
Analytical ABER and Monte Carlo simulation results (circles) vs. SINR $\gamma_{0}$ for a vw-MPPM format with code rate of 1/8 under weak ($\sigma_{h_{2}}^{2}=0.0496$), weak-to-moderate ($\sigma_{h_{2}}^{2}=0.2453$) and strong ($\sigma_{h_{2}}^{2}=1.0652$) salinity-induced turbulence and different Málaga turbulence intensities in the terrestrial FSO link. Scenarios and values of irradiance variances in the underwater medium taken from acquired data presented in [68]. In solid lines, the performance of the system when only oceanic turbulence is considered is represented.

This section is concerned with the ABER performance investigation of the proposed expressions provided in (21) and (25) for the cooperative AF-based dual-hop TU-FSO system. Most of the figures (Figure 3, Figure 4, Figure 5 and Figure 6) presented here offer the same information particularized for the concrete coding technique under analysis. Thus, in solid lines, the performance of the system when only oceanic turbulence is considered is represented; whereas the dashed, dashed-dotted, dotted or the line with a ‘+’ marker show the behavior of the system when including both oceanic and atmospheric turbulences. The corresponding Monte Carlo simulation results are displayed as circles. For all these cases and considering the approach employed in (12), we can assume that $\gamma_{0}$ can be seen merely as an SNR since ISI is included as an additional variance term previously averaged over oceanic turbulence.

Regarding oceanic turbulence and for the sake of clarity, we offer a correspondence between specific salinity-induced turbulence magnitudes, $\sigma_{h_{2}}^{2}$, employed in Figure 3, Figure 4, Figure 5 and Figure 6, and their associated underwater distance lengths. To this aim, we use a simple analytic approximation for the scintillation index proposed in Appendix A in [58], which is provided by the following.

(26) $$\sigma_{h_{2}}^{2}≃\lambda_{1}d^{2}+\lambda_{2}d+\lambda_{3},\;d\leq 100\;m.$$

In (26), the scintillation index parameters $\lambda_{1},$$\lambda_{2}$ and $\lambda_{3}$ were obtained after the curve-fitting approach. For the case of both plane wave and mostly salinity-induced turbulence, we can establish $\lambda_{1}=0.000536$, $\lambda_{2}=0.00507$ and $\lambda_{3}-0.013$, as summarized in Table 3 in [58]. Then, and for the values of $\sigma_{h_{2}}^{2}$ considered in Figure 3, Figure 4, Figure 5 and Figure 6 (0.0496, 0.2453 and 1.0652), we can obtain their associated underwater optical wireless communication link distances: d=7.07, 17.73 and 40.37 m, respectively. Namely, this fact means that, when the distance is 7.07 m, the scintillation index reaches a value of 0.0496; but the longer the distance, the stronger the intensity of turbulence and, for example, when d=40.37 m, $\sigma_{h_{2}}^{2}=1.0652$.

Nevertheless, these values of distance are obtained by assuming perfect transmitter–receiver alignment. In this respect, the total underwater fading coefficient is defined as two factors: $h=L\cdot h_{2}$, with *L* being the oceanic path loss, whereas $h_{2}$ denotes salinity-induced oceanic turbulence. Without a loss of generality and as commented in Section 2, our results, displayed in Figure 3, Figure 4, Figure 5 and Figure 6, did not consider the effects of absorption in water (nor the effect of weather-induced attenuation in the FSO link). Since oceanic absorption can be included as a pure loss term acting as a scaling factor, we offer an estimation to update the results, including *L* (geometric spread effect will not be considered). Thus, following the Beer–Lambert law as the classical exponential attenuation model, the intensity loss at a distance *d* is given by the following [58]:

(27) $$L=\exp\left(-Fc_{T}d\right),$$

where $c_{T}$ is the extinction coefficient, including absorption and scattering effects. On the other hand, *F* denotes the increase in received power due to scattering. The values for $c_{T}$ are provided in Table 1 in [58] for both clear ocean water and coastal water. If we suppose F=1, then, in that case, for the aforementioned distance values of d=7.07, 17.73 and 40.37 m, intensity loss equates to L=0.3438, 0.0688 and 0.0023 (−9.27 dB, −23.25 dB and −52.94 dB), respectively, when clear ocean water is considered. Whilst for coastal water, $L=5.997\times 10^{-2}$, $8.6175\times 10^{-4}$ and $1.052\times 10^{-7}$, again for d=7.07, 17.73 and 40.37 m, respectively. The penalties in dBs, in this latter scenario, are as follows: L=−24.44, −61.29 and −139.55 dBs, respectively. In this respect, it is straightforward to modify the x-axes corresponding to Figure 3, Figure 4, Figure 5 and Figure 6 to include the effect of absorption in water. For example, in Figure 3, the new x-axis will range from 28.25 to 73.25 dBs (instead of from 5 to 50) if either d=17.73 m and coastal water are considered, increasing the required SNR into 23.25 dBs to obtain the same values of BER once *L* is included.

Let us start with the uncoded OOK scheme, for which the results, shown in Figure 3 from the evaluation of Equation (21), will offer a reference for the other coded transmission techniques analyzed in this paper. As expected, for the lower intensity of turbulence, ABER tends to dramatically increase. These results show that in order to achieve an ABER of $10^{-4}$ in the presence of atmospheric and oceanic turbulence, it will be necessary to accomplish an SNR ranging from 11.5 dB (corresponding to a turbulence strength of $\sigma_{h_{2}}^{2}=0.0496$ for the underwater link and the absence of turbulence for the atmospheric one) to 50 dB (both terrestrial and underwater FSO paths characterized by a strong turbulence of $\sigma_{h_{1}}^{2}=2.699$ and $\sigma_{h_{2}}^{2}=1.0652$, respectively). In contrast, for the ideal case of the absence of turbulence in both terrestrial and the underwater media, only 8.6 dB is required for SNR to satisfy an ABER of $10^{-4}$.

Apart from that, from Figure 3, we may anticipate the preponderance of one stretch over the other according to the change presented in the slope of some ABER curves. In this respect, there may exist a higher vulnerability to one concrete segment (terrestrial or underwater) that can be evaluated from Equation (6). Hence, for high SNRs, $F_{\gamma_{1}}\left(\gamma \right)F_{\gamma_{2}}\left(\gamma \right)\approx 0$, and the CDF (and also the PDF) for the CSI-assisted AF-based TU-FSO system would be reduced to the sum of the CDFs (or PDFs) associated with terrestrial and underwater FSO hops. In this assumption, it is straightforward to determine which segment (terrestrial or submarine) contributes the most to the cooperative optical link. For example, in such Figure 3, the blue solid line represents the performance of the system when only oceanic turbulence is taken into account (let us remember that the scattering was introduced in the system as an average variance obtained after averaging the inter-symbol interference over the oceanic turbulence). On its part, the dashed-dotted purple line shows the behavior of the AF-based TU-FSO system for the ideal case of no oceanic turbulence and an atmospheric turbulence featured by α=50, β=10 and ρ=0.9. The inclusion of those two latter phenomena (dashed-dotted blue line) implies that the resulting performance is, approximately, the sum of the two single aforementioned PDF curves (only oceanic turbulence and only atmospheric turbulence). The same verification can be carried out with the solid red line (system with only oceanic turbulence of variance $\sigma_{h_{2}}^{2}=1.0652$) and the purple line with a ‘+’ marker (system with only Málaga atmospheric turbulence with α=1, β=2 and ρ=0.1).

On the other side and observing the results shown in Figure 3 in [69], we can observe how when ρ tends to 1, an improved performance of the entire system is obtained (when maintaining the values of α and β) since the overall atmospheric scattering power travels coupled to the LOS component; but when ρ→0, then all the scattering power travels through the $U_{S}^{G}$ field, which is statistically independent from the other field components, as represented in Figure 2. From Section 3, ρ denotes the amount of scattering power coupled to the LOS component.

As a last comment, the solid blue and red lines in Figure 3 (corresponding to $\sigma_{h_{2}}^{2}=0.0496$ and 1.0652, respectively, for the underwater FSO segment, while considering, in both cases, an ideal AWGN channel with an absence of turbulence for the terrestrial FSO link) will be subsequently displayed when showing the results associated with the coded transmission schemes (Figure 4, Figure 5 and Figure 6) with the purpose of providing an immediate comparison.

Regarding the case in which the CBER of any coding scheme is unknown and independent of itself, the hyperexponential fitting technique can be applied, resulting in a closed-form analytical expression for the ABER, as shown in Equation (25). There, by solely fitting the values for the parameters $a_{c},b_{c},$ and $c_{c}$ (see Table 1 for the schemes analyzed in this paper) in the ideal case of an AWGN channel (CBER), we can achieve a completely accurate performance for ABER. The inclusion of signaling techniques such as OOK-GScc and vw-MPPM and their behavior in Figure 4, Figure 5 and Figure 6 corroborates that feature. Hence, assuming the same previously considered value of ABER ($10^{-4}$) used with the aim of comparing all those coded transmission schemes with different code rates, the required SNRs to satisfy that aforementioned ABER are included in Table 2 for the different coding techniques addressed in this paper. Note that the values for the vw-MPPM format with a code rate of 1/4 are taken from Figure 2b in [44].

For all cases, vw-MPPM offers the best performance where codewords with different Hamming weight are permitted, obtaining higher improvements in performance as the length of the data block increases.

Finally, we want to remark that the accuracy of the proposed expressions in this paper is fully corroborated by numerical simulations, corroborating the validity of Equations (21) and (25).

## 6. Concluding Remarks

In this paper, the converging wireless optical communication system based on a cooperative terrestrial and underwater FSO scheme has been analyzed in terms of the average bit-error rate. The study here presented has considered the effects of the turbulence in both atmospheric and underwater environments into system performance, modeling the scintillation induced in each medium with Málaga and Weibull statistical distributions, respectively. Hence, the effects of both channels on the overall system behavior are highlighted with closed-form analytical expressions obtained to evaluate the ABER for uncoded and coded transmissions. Thus, in some of the results shown, it is possible to intuit the predominant effect of the terrestrial or submarine environment as a function of the signal-to-noise ratio. The analytical expressions here proposed also provide a simple and efficient procedure for estimating the behavior of any coding scheme for which the bit-error-rate behavior in an AWGN channel without turbulence can be adjusted by the hyper-exponential method employed here. It must be noted that the proposed closed-form expressions have been corroborated by Monte Carlo simulations for different turbulence conditions and several code rates. Remarkably, the new derived expressions resulted in a valuable tool for analyzing the performance of these cooperative optical links, which involved either the effects of the terrestrial and underwater channels or the behavior of different coding techniques.

## Figures and Tables

**Table 1:** Hyper-exponential fitting parameters $a_{c}$, $b_{c}$ and $c_{c}$ in the absence of turbulence.

Code	Code Rate	$a_{c}$	$b_{c}$	$c_{c}$
OOK-GScc	1/4	1.7103	30.2515	0.8592
	1/8	1.2465	120.1244	0.9386
vw-MPPM	9/36	0.7246	42.4424	0.8600
	5/40	1.1890	152.1918	0.8150

**Table 2:** SNR ($\gamma_{0}$) [dBs] for achieving an ABER of $10^{-4}$.

Code Rate	Terrestrial FSO Channel	Underwater FSO Channel	OOK-GScc	vw-MPPM
1/4	*Ideal AWGN ($\sigma_{h_{1}}^{2}=0$)*	$\sigma_{h_{2}}^{2}=0.0496$	0.75	−0.82
	$\sigma_{h_{1}}^{2}=2.699$	$\sigma_{h_{2}}^{2}=1.0652$	44	39.13
1/8	*Ideal AWGN ($\sigma_{h_{1}}^{2}=0$)*	$\sigma_{h_{2}}^{2}=0.0496$	−4.19	−4.15
	$\sigma_{h_{1}}^{2}=2.699$	$\sigma_{h_{2}}^{2}=1.0652$	40.06	36.8

## Data Availability

Not applicable.
